# Comparative analysis of chloroplast genome and evolutionary history of *Hemerocallis*

**DOI:** 10.3389/fgene.2024.1433548

**Published:** 2024-07-26

**Authors:** Jiang Wu, Yang Gao, Jinyao Wang, Aihua Guo, Nannan Qin, Guoming Xing, Sen Li

**Affiliations:** ^1^ College of Horticulture, Shanxi Agriculture University, Taiyuan, China; ^2^ Department of Life Science, Lyuliang University, Lüliang, China; ^3^ Datong Daylily Industrial Development Research Institute, Datong, China

**Keywords:** *Hemerocallis*, daylily, nightlily, chloroplast genome, ancestor state

## Abstract

Members of the genus *Hemerocallis* have significant value as ornamental, edible, and medicinal plants, particularly in China, where they have been utilized for thousands of years as both a vegetable and Traditional Chinese Medicine. *Hemerocallis* species exhibit strict control over flowering time, with individuals flowering either diurnally or nocturnally. However, our understanding of the evolutionary history of this genus, especially concerning important horticultural traits, remains limited. In this study, sequencing and assembly efforts were conducted on 73 samples within the *Hemerocallis* genus. All accessions were classified into two distinct groups based on their diurnal (daylilies) or nocturnal (nightlilies) flowering habits. Comparative analysis of the chloroplast genomes from these two groups identified fifteen variant hotspot regions, including fourteen SNPs and one deletion, which hold promise for the development of molecular markers for interspecific identification. Phylogenetic trees, generated through both maximum-likelihood and Bayesian inference methods using 76 shared protein-coding sequences, revealed that diurnal flowering evolved prior to nocturnal flowering. The divergence between the two groups is estimated to have occurred approximately 0.82 MYA (95% CI: 0.35–1.45 MYA). The ancestral state of *Hemerocallis* is hypothesized to have featured diurnal flowering with orange yellow petals. This study marks the first reconstruction of the evolutionary history and ancestral state of the genus *Hemerocallis*. The findings contribute significantly to our understanding of the adaptation and speciation history within the genus.

## 1 Introduction

The species of *Hemerocallis* (Asphodelaceae*:* Hemerocallidoideae) (Group, 2016) are mainly distributed in East Asia, with China as the center of origin. These species exhibit extraordinary morphological diversity, particularly in the flowers of hybrid cultivars, and have therefore been embraced by ornamental plant breeders around the world (Stout, 1934). The pre-blossom flower buds of *Hemerocallis citrina* Barni have been utilized as a fresh and dried vegetable for thousands of years in China (Qing et al., 2021). In addition, the genus is considered to possess exceptional medical value in Traditional Chinese Medicine (Wang et al., 2018; Ma et al., 2023; Zhao et al., 2024).

All members of genus *Hemerocallis* exhibit two distinct features. The first is strict control over flower opening time, with flowers opening either diurnally or nocturnally (Kang and Chung, 2000; Hasegawa et al., 2006; Ren et al., 2021). The second is that the blossom duration for individual flowers never exceeds 24 h (Rodriguez-Enriquez and Grant-Downton, 2013; Ren et al., 2021; Yang et al., 2021). These peculiarities have earned the daytime flowering members the moniker “daylily,” and likewise, the nighttime flowering members the moniker “nightlily” (Li et al., 2021). Among the 11 wild *Hemerocallis* species native to China, eight bloom diurnally (*Hemerocallis fulva*, *H. esculenta*, *H. nana*, *H. dumortieri*, *H. middendorfii*, *H. multiflora*, *H. plicata*, and *H. forrestii*) and three bloom nocturnally (*H. lilioasphodelus*, *H. citrina*, and *H. minor*) (Hirota et al., 2021). In general, diurnal daylilies bloom between 4:30 and 7:30 UTC, while nocturnal nightlilies bloom between 16:30 and 20:30 UTC (Hasegawa et al., 2006).

Because both groups bloom during completely different intervals, they are preferentially pollinated by different insects (Hasegawa et al., 2006; Yasumoto and Yahara, 2006). Despite this, the two groups are not reproductively isolated, and fertile F1 generations can be produced following pollination (Stout and Chandler, 1933; Kawano, 1961; Stout, 1986; Hasegawa et al., 2006). Research into the evolutionary history of genus *Hemerocallis* suggests that the differentiation of flowering time-associated traits may be an internal mechanism for maintaining genetic diversity. Variation in flowering time is directly related to environmental adaptation, and therefore understanding the molecular mechanism underlying variation in flowering time is crucial for understanding *Hemerocallis* adaptation, reproductive isolation, and domestication (Stinchcombe et al., 2004; Kollmann and Bañuelos, 2004; Kawakami et al., 2011; Dijk and Hautekèete, 2014; Gaudinier and Blackman, 2019). However, the whole evolutionary history of genus *Hemerocallis* is still poorly resolved, particularly those events pertaining to the development of important traits.

Chloroplasts, key centers of photosynthesis and biosynthesis within plant cells, are semi-autonomous organelles possessing autonomous genetic systems (Sugiura, 1995). Current evidence suggests that chloroplasts originated from endosymbiotic cyanobacteria (Zhang et al., 2023). In most angiosperms, the chloroplast genome has a low recombination rate and is passed to the next-generation as a complete haplotype. Compared to the nuclear and mitochondrial genomes, the chloroplast genome exhibits a moderate nucleotide replacement rate. Due to these characteristics, the chloroplast genome is well-suited for phylogenetic reconstruction and evolutionary route tracing, particularly among closely-related species or members of the same population. Over the past decade, the chloroplast genome has become an important resource for phylogenetics and divergence time estimation (Sugiura, 1995).

A phylogenetic tree that accurately represents the relationships among members of the genus *Hemerocallis* is crucial for comprehending the origin and evolutionary history of important traits. In this study, we utilized the chloroplast genome to construct phylogenetic relationships within the genus *Hemerocallis*, aiming to elucidate the relationships among branches. Subsequently, we examined the variations and hotspot mutation regions among groups. Additionally, we identified differentiation events and reconstructed the ancestral traits of *Hemerocallis*, highlighting the importance of adaptation associated with flowering time during the evolutionary history of this genus. The findings of this study will enhance our understanding of the evolutionary trajectory of *Hemerocallis* and holds significance for the genetic enhancement and breeding of germplasm resources.

## 2 Materials and methods

### 2.1 Sample collection, DNA extraction, and sequencing

All materials used in this study were collected from plants cultivated at the experimental orchard located at Shanxi Agriculture University. The majority of the samples were sourced from China, with some originating from other countries and regions. Due to the inherent instability and inconsistency of horticultural traits in newly-transplanted plants, all re-sequenced accessions were selected from plants that had been transplanted for more than 3 years and exhibited stable and consistent traits. After years of phenotypic evaluation and statistical analysis, a total of 73 accessions were selected (Supplementary Table S1). Genomic DNA was extracted using the plant genomic DNA kit (TaKaRa, Kusatsu, Japan). DNA quality was assessed using a Nanodrop 2000 (Thermo Scientific, Waltham, MA, United States) and by 1% (w/v) agarose gel electrophoresis. Libraries were constructed and sequenced in 150 bp paired-end reads using the Illumina HiSeq 2000 platform at the Genedio Company (Guangzhou, China).

### 2.2 Assembly and annotation of the chloroplast genome

For each sample, approximately 1G of reads were extracted from the clean data to assemble the chloroplast genome. We employed GetOrganelle 1.7.5 (Jin et al., 2018) for *de novo* assembly with recommended parameters, using our previously assembled chloroplast genome as reference (NC_064967.1). Novowrap (Wu et al., 2021) was utilized to adjust the direction of the inverted repeat (IR) region and the starting site of the newly assembled genome. To validate circularization of assembled sequences, Bandage v0.9.0 (Wick et al., 2015) was employed. In cases of incompletely assembled genomes, SAMtools v1.20 (Danecek et al., 2021) was used to obtain the bam file at the breakpoint to further verify the read depth. Subsequently, the chloroplast genomes were submitted to CPGAVS2 (Shi et al., 2019) for annotation, combined with manual corrections by Geneious Prime v2021. Annotation of tRNAs was conducted using tRNAscan-SE v2.0 (Chan et al., 2021). The complete chloroplast genome was visualized using OGDRAW v1.3.1 (Lohse et al., 2007). All chloroplast genomes were deposited in GenBank (Supplementary Table S1).

### 2.3 Phylogenetic analysis

The phylogenetic tree was constructed using the whole chloroplast genome. To avoid impacts resulting from poor alignments caused by gaps, the intergenic sequences between *trnT-UGU* and *trnL-UAA* were removed from all sample. Then samples with identical genome sequences were filtered, retaining only one representative for further analysis. Subsequently, the sequences were aligned using Mafft v7.487 (Katoh et al., 2002). The dataset were inspected manually and automatically trimmed using trimAl v2.0 (Capella-Gutierrez et al., 2009). jModeltest v2.1.10 (Darriba et al., 2012) was employed to identify the best nucleotide evolution model, selected based on the Akaike Information Criterion (AIC). IQ-TREE2 (Minh et al., 2020) was utilized to build the ML tree, specifying the molecular substitution models GTR + I + G, with the bootstrap set to 1,000 cycles. The final result was visualized using FigTree (http://beast.bio.ed.ac.uk/FigTree).

### 2.4 Comparison analysis between groups

The IRscope (Amiryousefi et al., 2018) online tool was employed to analyze and visualize the contraction and expansion of the IR regions and the SSC (short single copy) region. Variant hotspot regions were identified by aligning all samples using mVISTA v2.0 (Frazer et al., 2004) with the LAGAN (Brudno et al., 2003) alignment program, using S129 as the reference sequence. Nucleotide polymorphism was computed by DnaSP v6.12.03 (Rozas et al., 2017), with window and step sizes set to 500bp and 50bp, respectively. The results were visualized using R. To further analyze the distribution of variants between the two clades, Geneious was utilized to find the location of all variants and calculate the variant counts among different samples.

### 2.5 Reconstruction of evolutionary relationships

In order to avoid interference resulting from interspecific hybridization, we developed a new dataset consisting of nine representative samples belonging to six *Hemerocallis* species. Four *Asparagales* species served as outgroups. Phylogenetic relationships were evaluated based on whole-genome sequences. With the inclusion of outgroups, after aligning the sequences in MAFFT, the Phylosuite v1.2.3 (Zhang et al., 2020) module “treesuite” was utilized for sequence substitution saturation analysis to assess the suitability of the data for phylogenetic reconstruction. Both the maximum-likelihood (ML) and bayesian inference (BI) methods were used, and analysis of the ML tree was the same as that of the unrooted tree. Bayesian phylogenetic analysis was executed using MrBayes v3.1.2 (Ronquist et al., 2012), applying the best fit model identified according to the AIC as determined by jModeltest2. The Markov Chain Monte Carlo (MCMC) analysis was run for 1,000,000 generations, with trees sampled every 1,000 generations, “lset nst” set to “6,” “rates” set to “invgamma,” and two MCMC chains run independently. Each chain began with a random tree with default prior values and was sampled every 100 generations, with the top 25% discarded as burn-in. The operation was deemed stable and then terminated automatically when the aggregate diagnostic value reached ≤ 0.01 (stopval = 0.01). Tracer (Lee et al., 2021) was used to evaluate convergence. Both ML and BI trees were visualized using Figtree.

### 2.6 Divergence time analysis

MCMCtree was employed in PAML v4.7 (Yang, 2007) to estimate the speciation time among members of *Hemerocallis*. The topological structure obtained as described above served as the input tree file. Given the absence of a fossil record for the study species, the molecular clock was calibrated twice based on information from the TimeTree database (http://www.timetree.org/) (Kumar and Hedges, 2017). Initially, the tree was calibrated by fixing the crown origin at 71.9 MYA. Subsequently, the occurrence of *Asphodelaceae* was constrained to the time range of 52.2–67.4 MYA.

### 2.7 Ancestral reconstruction

In order to reconstruct the ancestral status of *Hemerocallis*, we retained the tree derived from BI analysis to infer the flowering time and petal color of the common ancestor. The maximum parsimony (MP) method was conducted in Mesquite v3.81 (Chung-hau et al., 2006) to reconstruct the evolutionary history of the two phenotypic traits. The character states were treated as unordered and coded as follows: 0, yellow; 1, golden; 2, orange yellow; 3, reddish orange; 0, daylily; 1, nightlily. The data for each morphological trait were obtained from both published literature (Li et al., 2021) and the field observations conducted by our research group. The data matrix can be found in Supplementary Table S8.

## 3 Results and discussion

### 3.1 General features of the *Hemerocallis* chloroplast genome

**FIGURE 1:**
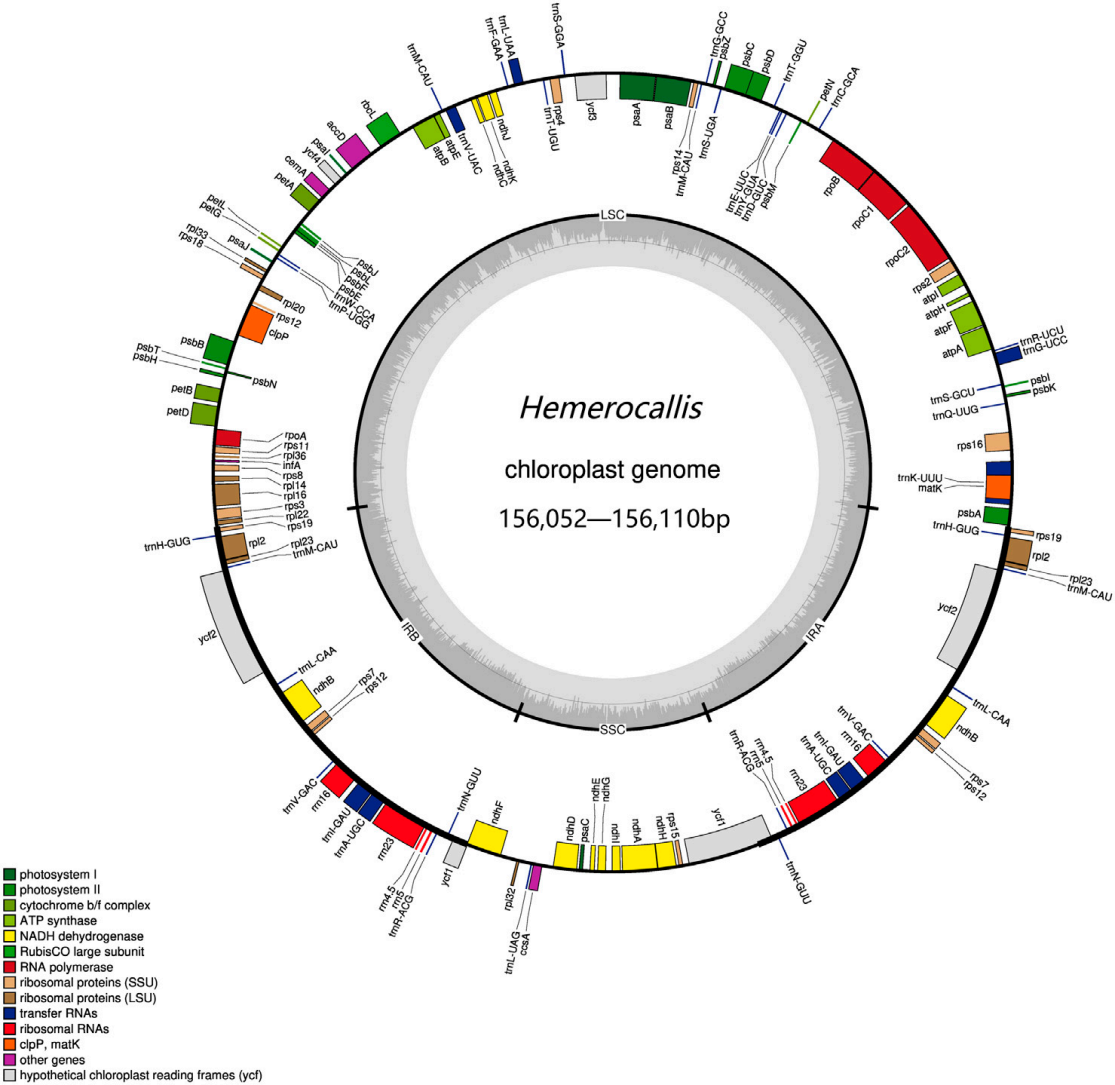
The *Hemerocallis* chloroplast genome. On the outer circle, the inner genes were transcribed counterclockwise and the outer genes were transcribed clockwise. On the inner circle, the dark gray bars indicated the GC content.

Of the 73 samples assembled, 26 resulted in complete chloroplast genomes while 47 remained uncyclized. We used Bandage to visualize the imcomplete genome and found that all samples exhibited discontinuities in the large single copy (LSC) region. Further examination of the annotation files revealed that gaps in the chloroplast genome were consistently appearing in the *trnT-UGU* and *trnL-UAA* intergenic region. The complete chloroplast genomes ranged in length from 156052 to 156102 base pairs (bp) (Figure 1). The LSC regions varied from 18501 to 18531bp, the SSC regions ranged from 84804 to 84843bp, and IR measured 26369bp in length (Supplementary Table S3).

The *Hemerocallis* chloroplast genome contains 110 genes, including 78 protein-coding, 4 rRNA, and 28 tRNA genes. Among these, 19 genes were duplicated in the inverted repeat regions, encompassing eight tRNA, four rRNA, and seven protein-coding genes. Nine genes (*atpF, rpoC1, petB, petD, rps16, trnG-UCC, trnK-UUU, trnL-UAA, and trnV-UAC*) contain a single intron, while two genes (*clpP* and *ycf3*) contain two introns (Supplementary Table S2). No gene rearrangements were observed in any of the chloroplast genomes (complete and incomplete), and the number of genes remained relatively conserved across samples. The overall guanine-cytosine (GC) content ranged from 37.21% to 38.45%, with the LSC region containing 35.08% GC, the SSC region containing 31.96% GC, and the IR region containing 42.87% GC (Supplementary Table S3). Overall, these results are consistent with previous reports (Jia et al., 2024).

### 3.2 Phylogenetic relationships among *Hemerocallis*

**FIGURE 2:**
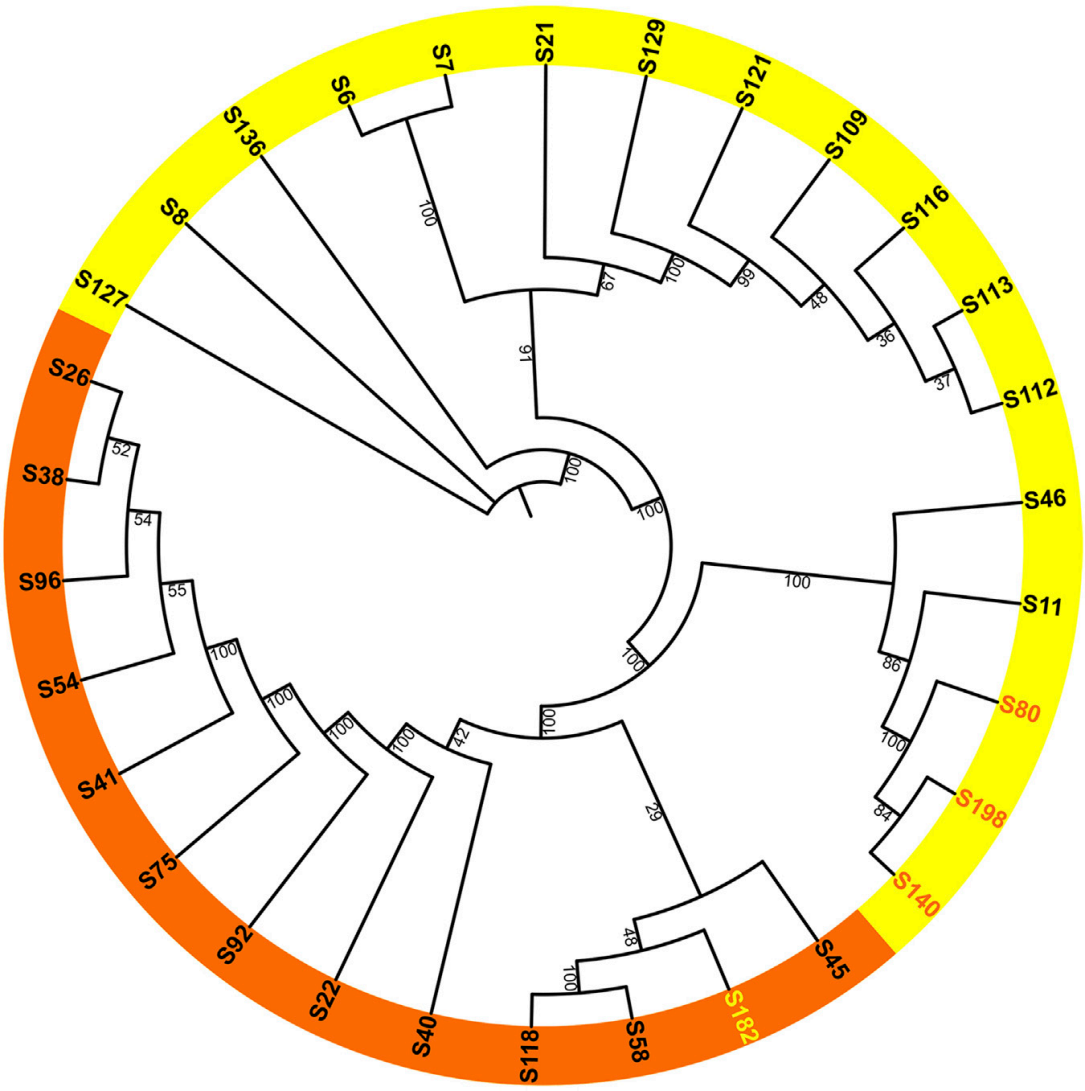
Maximum-likelihood phylogenetic reconstruction of 30 *Hemerocallis* accessions based on chloroplast genomes. The daylilies are highlighted in orange, while the nightlilies are highlighted in yellow. Bootstrap values are derived from 1,000 bootstrap replicates.

After removing duplicate genome sequences (Supplementary Table S4), a total of 30 samples remained. An unrooted ML trees and BI tree were constructed using the whole chloroplast genome as input data, and the main branch bootstrap values were all greater than 90 (Figure 2). Considering the trait of flowering time, the phylogenetic tree can be broadly divided into two groups, with most samples within each group exhibiting consistent flowering characteristics. The diurnal flowering group included *H. fulva* (S26), *H. middendorffii* (S41), *H. multiflora* (S45), and *H. fulva var. aurantiaca* (S40). Meanwhile, the nocturnal flowering group included *H. lilioasphodelus* (S8), *H. minor* (S7), *H. citrina var. altissima* (S46) and *H. citrina* (S6, S128, etc.). Notably, the diurnal flowering group also included a nocturnal flowering samples (S182), while the nocturnal flowering group included three diurnal flowering sample (S140, S40, and S198). Among these four samples, all of them were cultivars.

### 3.3 Comparative analysis of Cp genome

**FIGURE 3:**
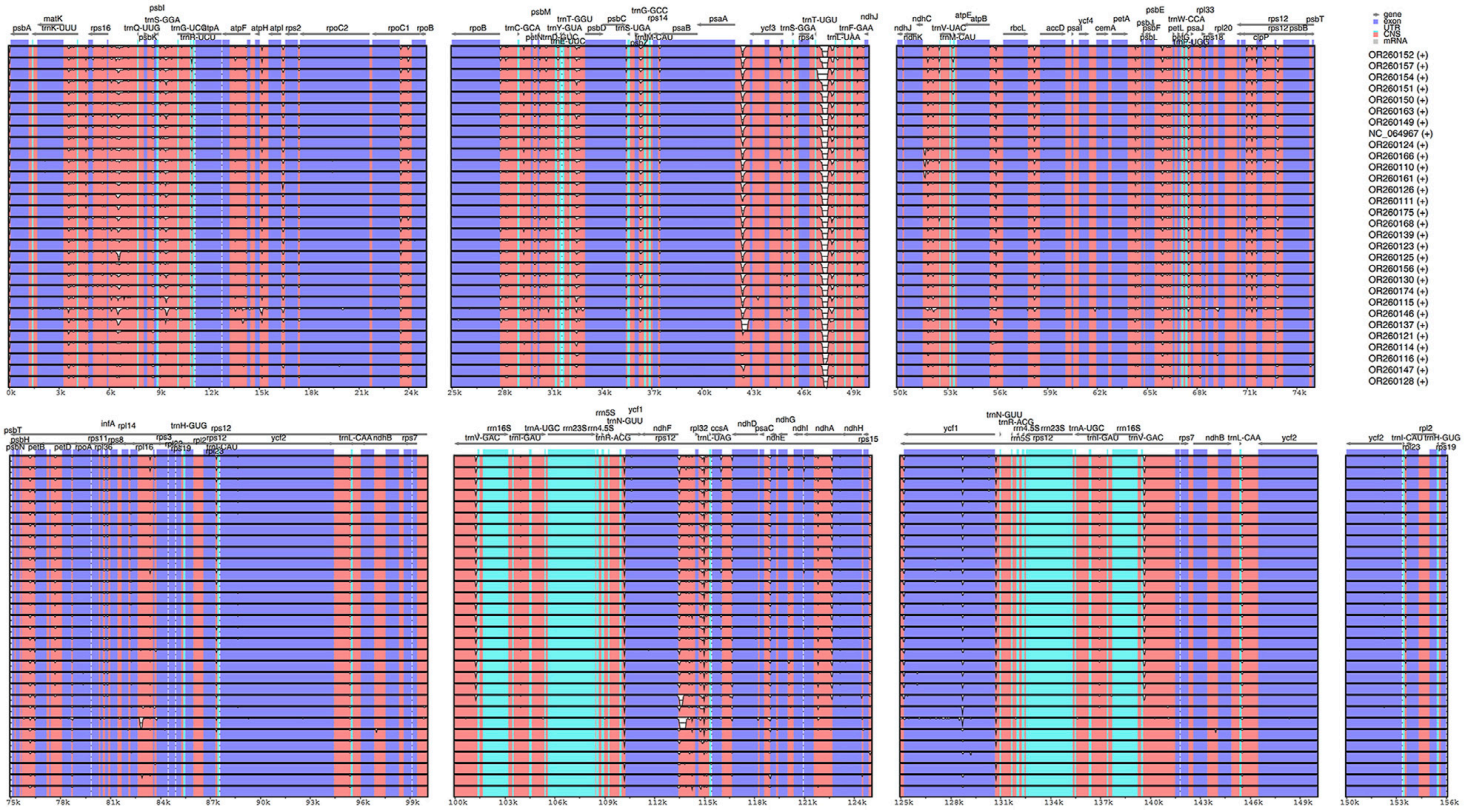
Sequence alignment of the plastome sequences of 30 *Hemerocallis* samples with OR260163 (S129) as reference. The gray arrows indicate the direction of genes. Blue and white correspond to coding regions and non-coding regions, respectively.

**FIGURE 4:**
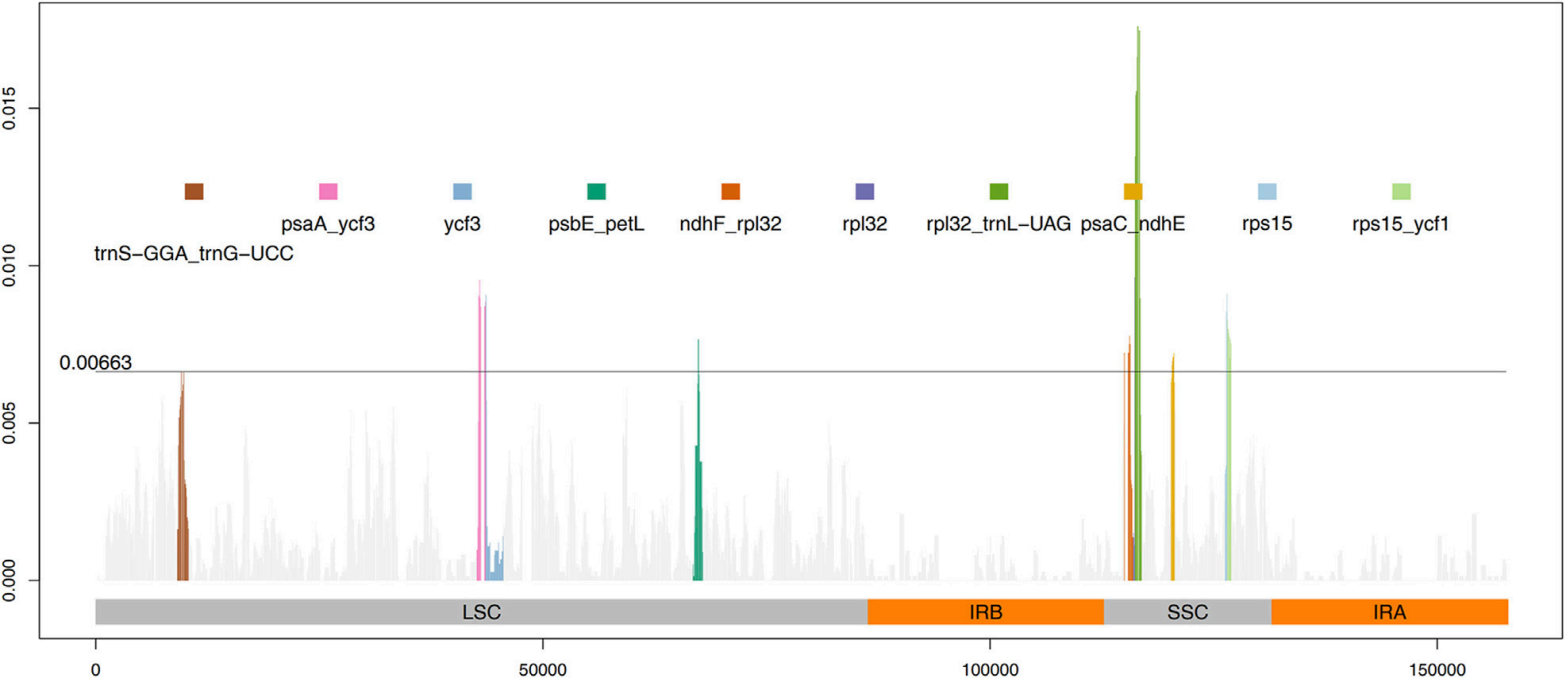
Sliding window calculation of nucleotide diversity (Pi) in the *Hemerocallis* chloroplast genomes with window width 500 bp and step size 50 bp. The X-axis represents the position of the midpoint of a window, and the Y-axis represents the nucleotide diversity of each window. The threshold line indicates the 10th peak of Pi value.

The results from IRscope (Supplementary Figure S1) analysis indicated that there were no significant differences in the lengths of the IRs and SSC regions between daylilies and nightlilies. The length difference of the *ycf1* gene located at the IRa and SSC boundary region was minimal, while the lengths of the *ycf1* and *ndhF* genes located at the IRb and SSC boundary region were identical between the two groups. Examination of the results from mVISTA revealed that non-coding regions of *Hemerocallis* chloroplast genomes exhibited higher polymorphism than coding regions, consistent with observations in most plants (Figure 3). According to calculations from DnaSP, the IR region being the most conservative (Pi value: 0.000361), the SSC region exhibiting the highest polymorphism (Pi value: 0.002577), and the LSC region having a medium level of polymorphism (Pi value: 0.001582). Additionally, the figure displayed the top ten nucleotide polymorphism sites on the genome, which served as hotspot regions of variation. Among them, seven peaks were located in intergenic regions (*trnS-GGA_trnG-UCC, psaA_ycf3, psbE_petL, ndhF_rpl32, rpl32_trnL-UAG, psaC_ndhE, rps15_ycf1*), while the remaining three peaks were located within gene sequences (*ycf3, rpl32, rps15*) (Figure 4; Supplementary Table S5). Based on the annotation file of the reference genome, fifteen variants specific to the Daylily group were identified, including fourteen SNP variants and one deletion (Supplementary Table S6). These variant sites could be further developed as molecular markers for distinguishing.

### 3.4 Evolutionary relationships among members of *Hemerocallis*

Both ML and BI trees were constructed using 76 shared protein-coding sequences (Supplementary Figure S3). Overall, the phylogenetic topologies obtained using the two methods were essentially identical, with high support at most nodes. *Hemerocallis* and *Xanthorrhoea,* two genera in subfamily Hemerocallidoideae, first clustered together and then clustered with members of family Asphodelaceae. The genus of *Hemerocallis* contained two sub-groups: daylily and nightlily. The daylily group compromised *H. fulva* (S27) and its variety (S40), *H. multiflora* (S45), and *H. middendorfii* (S41), which all exhibited daytime flowering. On the other hand, the nightlily group consisted of *H. lilioasphodelus* (S8), *H. minor* (S7) and two cultivars of *H. citrina* (S6, S128), all of which bloom at night. Meanwhile, the daylily was positioned closer to the root. These results suggest that nightlilies were derived from daylilies.

### 3.5 Divergence history of the genus *Hemerocallis*

**FIGURE 5:**
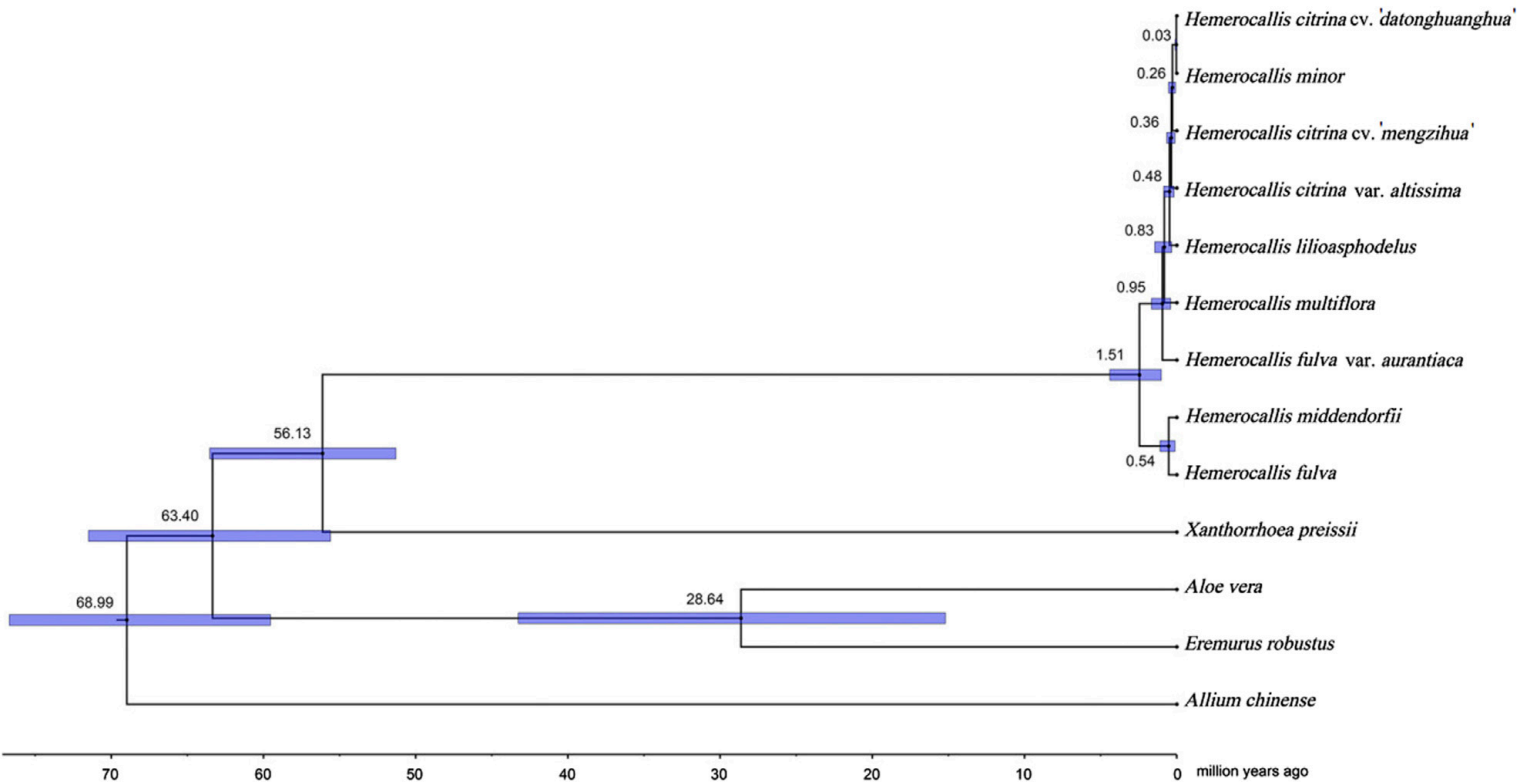
*Hemerocallis* divergence time estimation based on Bayesian inference tree. Divergence time analysis was conducted using MCMCtree. All estimates are shown with their 95% confidential intervals (95% CI) highlighted in blue.

In order to reconstruct the divergence history of the two *Hemerocallis* groups, we performed a time divergence analysis based on a phylogenetic tree with multiple calibrations (Figure 5). The MCMCtree results revealed that the common ancestor of *Hemerocallis* occurred during the late Paleocene, approximately 56.13MYA (95% CI: 51.31–63.54 MYA). Differentiation occurred gradually during the second stage of the Miocene, approximately 19.2 MYA, which was somewhat earlier than previously reported (Hirota et al., 2021). The ancestor split into the daylily and nightlily at approximately 0.82 MYA (95% CI: 0.35–1.45MYA). Notably, the daylilies emerged earlier than the nightlilies. The divergence of multiple species within *Hemerocallis* mainly occurred within the time range approximately 1.92–0.03MYA. This result is fairly consistent with previously report (Hirota et al., 2021).

### 3.6 Ancestral state of *Hemerocallis*

**FIGURE 6:**
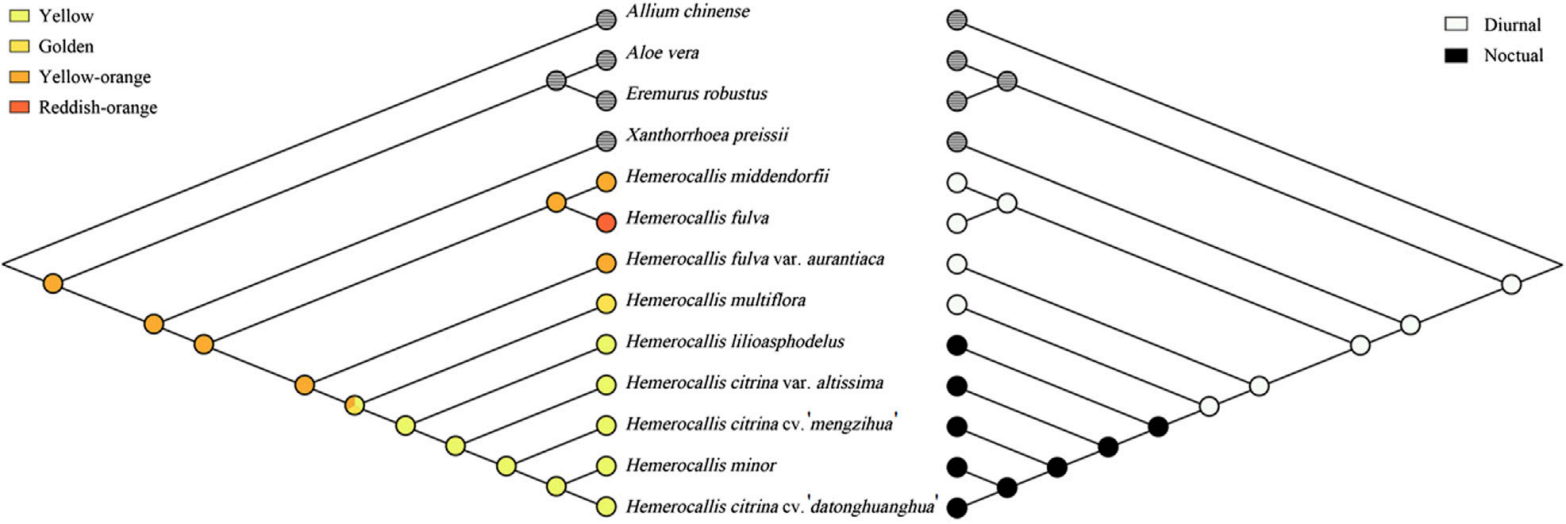
Ancestral states of petal color (left) and flowering time (right). Trees were reconstructed in Mesquite using the maximum parsimony method. Each trait was coded as follows: 0, yellow; 1, golden; 2, orange yellow; 3, reddish orange; 0, daylily; 1, nightlily. Traits for outgroups were left blank.

To further investigate population differentiation within *Hemerocallis*, we explored evolutionary trends associated with the flowering time and petal color traits (Figure 6). The ancestral state was reconstructed using the Cp genome-based BI tree (Supplementary Figure S3). The ancestor of *Hemerocallis* most likely had orange yellow petals and flowered diurnally, suggesting that nocturnal flowering and yellow evolved later.

## 4 Discussion

The *Hemerocallis* chloroplast genome was relatively conserved, characterized by a typical quadripartite structure consisting of LSC region, one SSC region, and two IRs. In all incompletely assembled genomes, breakpoints occurred at the same position. This outcome was primarily attributed to the GC bias of certain second-generation sequencing platforms (Browne et al., 2020). According to statistical analysis, the GC content in the LSC region of *Hemerocallis* chloroplast genomes was notably low (17.27%–17.44%) (Supplementary Table S3), resulting in low coverage of reads in this region and leading to gaps. Similar occurrences have also been noted during the assembly of chloroplast genomes in other species (Xue et al., 2012; Wang et al., 2013; Chen et al., 2023b).

Modern *Hemerocallis* cultivars have originated from hybridization among a limited number of species, which subsequently served as the parental lines for many ornamental cultivars (Tomkins et al., 2001). Due to the maternal inheritance of chloroplast genomes in *Hemerocallis*, and flowering time is controlled by genes of the nuclear genome (Chaves-Silva et al., 2018; Sunil and Shetty, 2022), classification based on flowering time traits may not align perfectly with molecular sequences. For instance, in our study, some cultivars (e.g., S198, S140, S40, and S182) were all misclassified when considering only chloroplast data, suggesting the need to combine nuclear genome data for accurate classification.

Intensive breeding has significantly reduced genetic diversity (Rodriguez-Enriquez and Grant-Downton, 2013). In this study, the Pi value of the whole chloroplast genome for all samples was only 0.00128, further confirming the narrowing of genetic diversity due to artificial selection pressure. Despite the high variability in floral traits among cultivars, they shared the same chloroplast genome (Supplementary Figure S4), a trend particularly evident in ornamental varieties. However, the phylogenetic structure generally aligns with flowering time traits, further supporting the rationality of subgrouping *Hemerocallis* into daylilies and nightlilies after removing hybrid species interference (Supplementary Figure S3).

Comparative analysis revealed no significant differences in the IR/SSC boundary positions between daylilies and nightlilies, suggesting minimal evolutionary shifts in these regions. Non-coding regions exhibited higher polymorphism compared to coding regions, consistent with most plants (Chen et al., 2023a; Li et al., 2024). Additionally, fifteen variations showed significant differences between daylilies and nightlilies. According to the reference genome annotation file, four SNPs were located within genes (*ycf1, ycf2, ycf4*) (Supplementary Table S6), indicating that these genes might have undergone strong selection pressure over the course of evolution. These variations might have a critical influence on the flowering time in *Hemerocallis*. These variations could serve as potential molecular markers for the identification of *Hemerocallis* species, aiding the breeding of new *Hemerocallis* cultivars with desired traits, and assisting in conservation efforts by identifying genetic diversity hotspots.

Our study included most wild species within the *Hemerocallis*, providing a relatively accurate reflection of the phylogenetic relationship between daylilies and nightlilies. ML and BI trees shared the same topology, indicating that nightlilies evolved from daylilies. These findings are consistent with previous results (Hirota et al., 2021). Continuous variation in external morphology among different species complicates classification, as seen with *H. lilioasphodelus*, *H. minor*, and *H. citrina*. They share highly similar external morphologies and karyotypes, leading some to suggest treating *H. minor* and *H. citrina* as two subspecies of *H. lilioasphodelus* (Zhi-Ting et al., 1996; Zhi-Ting and Singchi, 1997). Our topology suggests that *H. lilioasphodelus* appeared first within the nightlily group. However, the evolutionary relationship between *H. minor* and *H. citrina* cv. *‘datonghuanghua’* was closer, with both appearing around 0.23MYA, later than *H. citrina* cv. *‘mengzihua’*. *H. citrina* cv. *‘mengzihua’* is a well-known landrace of *H. citrina* in Hunan Province, China, while *H. citrina* cv. *‘datonghuanghua’* is another landrace in Shanxi Province, China, both with a long cultivation history. According to literature records, wild *H. citrina* is mainly distributed south of the Qinling Mountains, while wild *H. minor* is more prevalent in northern and northeastern China (Wu, Raven, and Hong, 2000). Therefore, we speculate that *H. citrina* cv. *‘datonghuanghua’* might originally belong to *H. minor*, although this conjecture required further verification.

The divergence time analysis indicated a rapid species expansion during the Pleistocene, a period characterized by significant climatic fluctuations. These environmental changes likely exerted selective pressures that drove trait divergence within the genus. For example, the shift from diurnal to nocturnal flowering may have been a strategy to avoid competition for pollinators or to exploit different pollinator species active at night. This adaptive flexibility would have been advantageous in the face of varying Pleistocene climates, promoting speciation and diversification within the genus.

Reconstructing the evolutionary history of *Hemerocallis* is crucial for understanding key traits. Our study utilized outgroups to root the phylogenetic tree and inferred that the ancestral state featured orange-yellow petals and diurnal flowering. This ancestral trait set provides significant insights into the adaptive strategies and ecological niches occupied by early *Hemerocallis* species.

However, our current understanding is based on a limited number of species, which constrains the robustness of our conclusions. The limited sampling could overlook key variations and evolutionary events that are critical to fully understanding the evolutionary trajectory of *Hemerocallis*. For instance, incorporating more wild species, especially those from underrepresented regions or habitats, could reveal additional adaptive traits and evolutionary pathways. Furthermore, integrating data from nuclear genomes alongside chloroplast genomes could provide a more comprehensive view of the evolutionary dynamics. Nuclear genomes, which are subject to different selective pressures and inheritance patterns compared to chloroplast genomes, can offer insights into gene flow, hybridization events, and the role of polyploidy in the evolution of *Hemerocallis*. Future studies should also consider the ecological interactions between *Hemerocallis* species and their pollinators, herbivores, and symbiotic partners. Understanding these interactions can shed light on the selective pressures that have shaped the evolution of key traits.

## 5 Conclusion

In this study, we selected a total of 74 samples to construct the phylogenetic relationships, further confirming the division of *Hemerocallis* into daylilies and nightlilies. Comparative analysis of the chloroplast genomes of the two groups revealed a relatively conserved chloroplast structure and boundary regions of the two IRs. Fifteen variant sites were identified, which showed group-specific characteristics and could serve as molecular markers for identifying daylilies and nightlilies in future studies. The phylogenetic relationships with outgroups not only reaffirmed the earlier appearance of the daylily group compared to the nightlily group but also indicated that *Hemerocallis* underwent evolutionary radiation in its long history. The ancestral state was inferred to feature diurnal flowering and orange yellow petals. To our knowledge, this is the first prediction of the ancestral state of *Hemerocallis*, and the identification of molecular markers for the two groups can also provide a basis for molecular breeding.

## Data Availability

The datasets presented in this study can be found in online repositories. The names of the repository/repositories and accession number(s) can be found in the article/Supplementary Material.
