# Supplementary material for: Comparative analysis of chloroplast genome and evolutionary history of Hemerocallis
Source: Front Genet. 2024 Jul 26;15:1433548. doi: 10.3389/fgene.2024.1433548 (PMC11310003; doi:10.3389/fgene.2024.1433548)
Supplement: Supplementary file 1 [file Table1.DOCX]

Supplementary Material

# Supplementary Figures


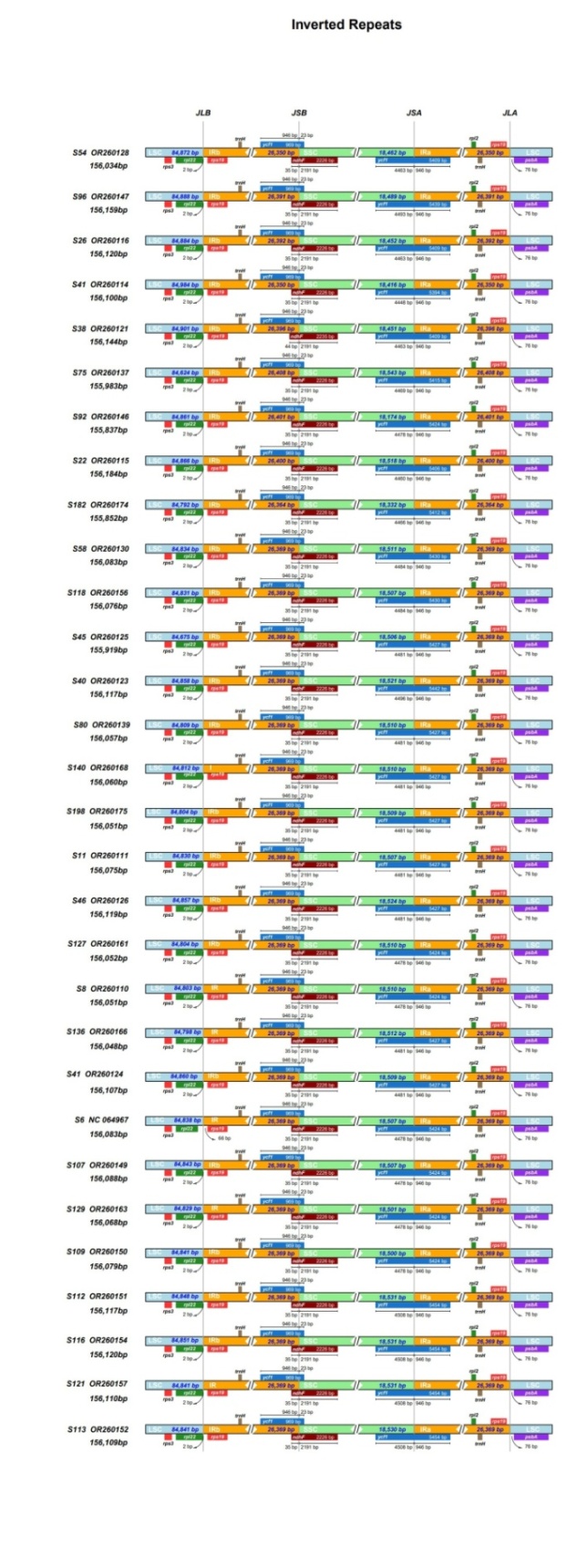


**Fig. S1** Comparison of the junctions between the LSC/SSC and IR regions among the 30 non-redundant *Hemerocallis* chloroplast genomes by IRscope.


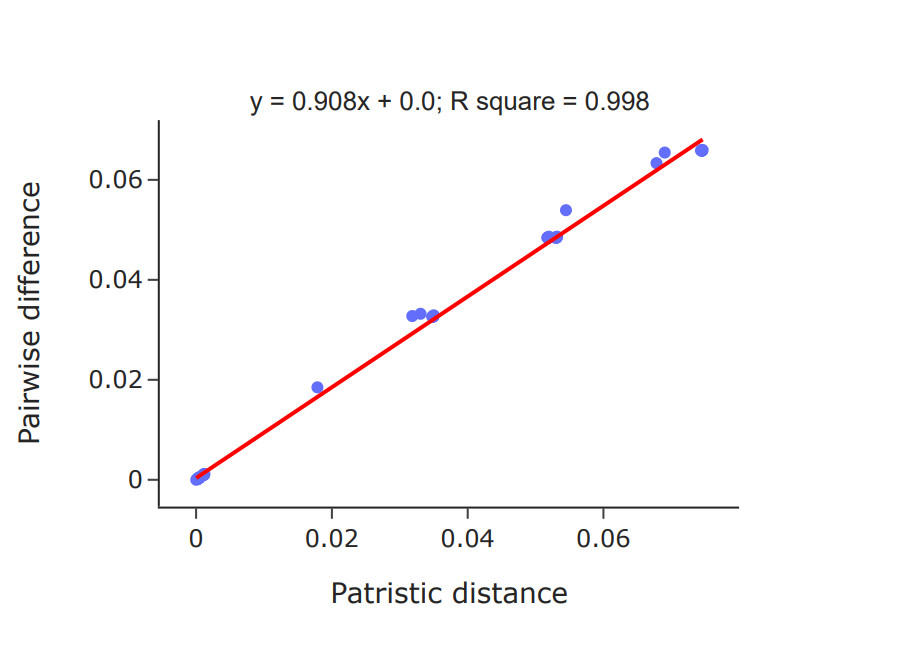


**Fig. S2** Saturation regression curve for the phylogenetic reconstruction following the inclusion of outgroups. Among the samples, 9 were from *Hemerocallis* and the remaining 4 were outgroups. The sequence matrix consisted of 76 CDS sequences. The R-square value was close to 1, indicating that the sequence was not saturated.


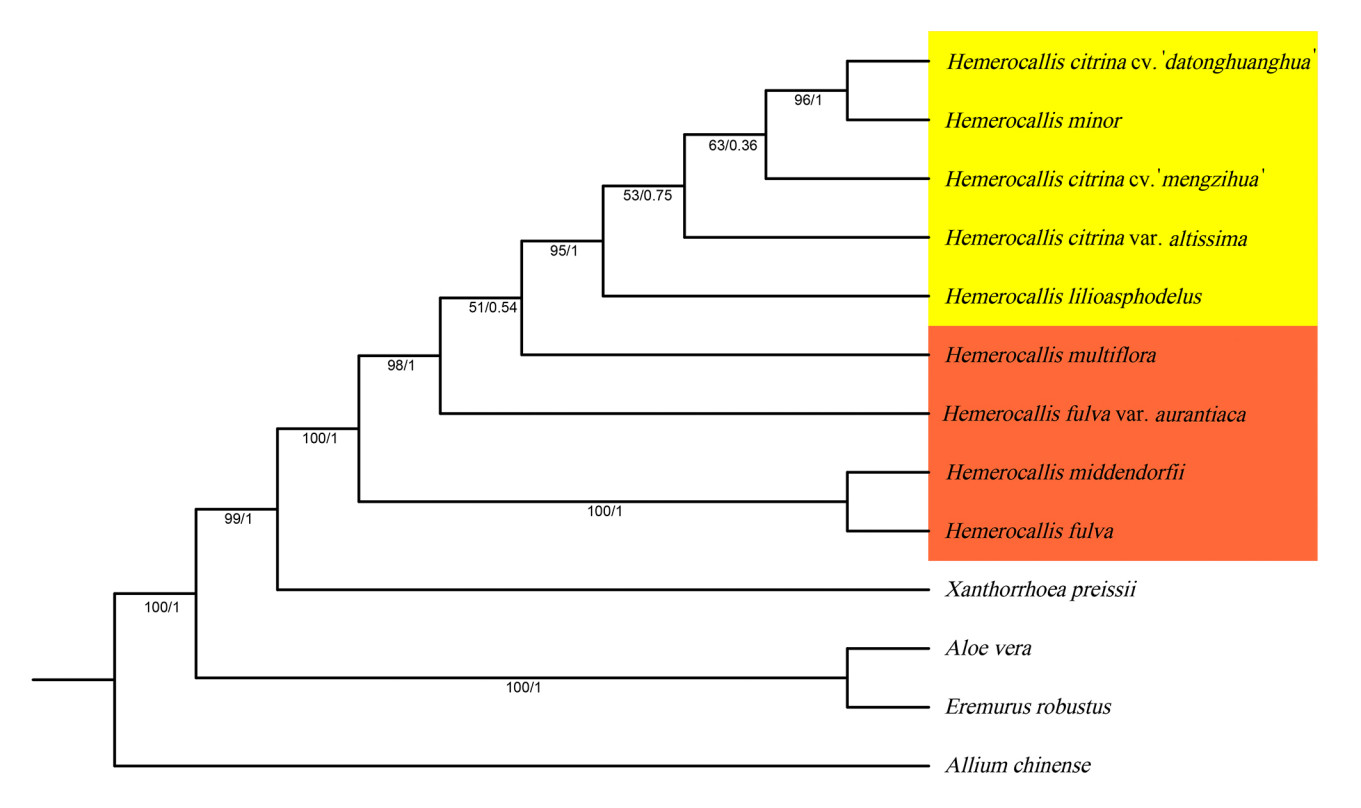


**Fig. S3** Phylogenetic tree based on 76 coding protein sequences inferred using maximum-likelihood (ML) and Bayesian inference (BI) methods. *Allium chinense* was set as the outgroup. The daylilies are highlighted in orange, while the nightlilies are highlighted in yellow. The numbers above the branches represent support values, with ML bootstrap values on the left and Bayesian bootstrap values on the right. Branches with bootstrap values below 50 are not shown.


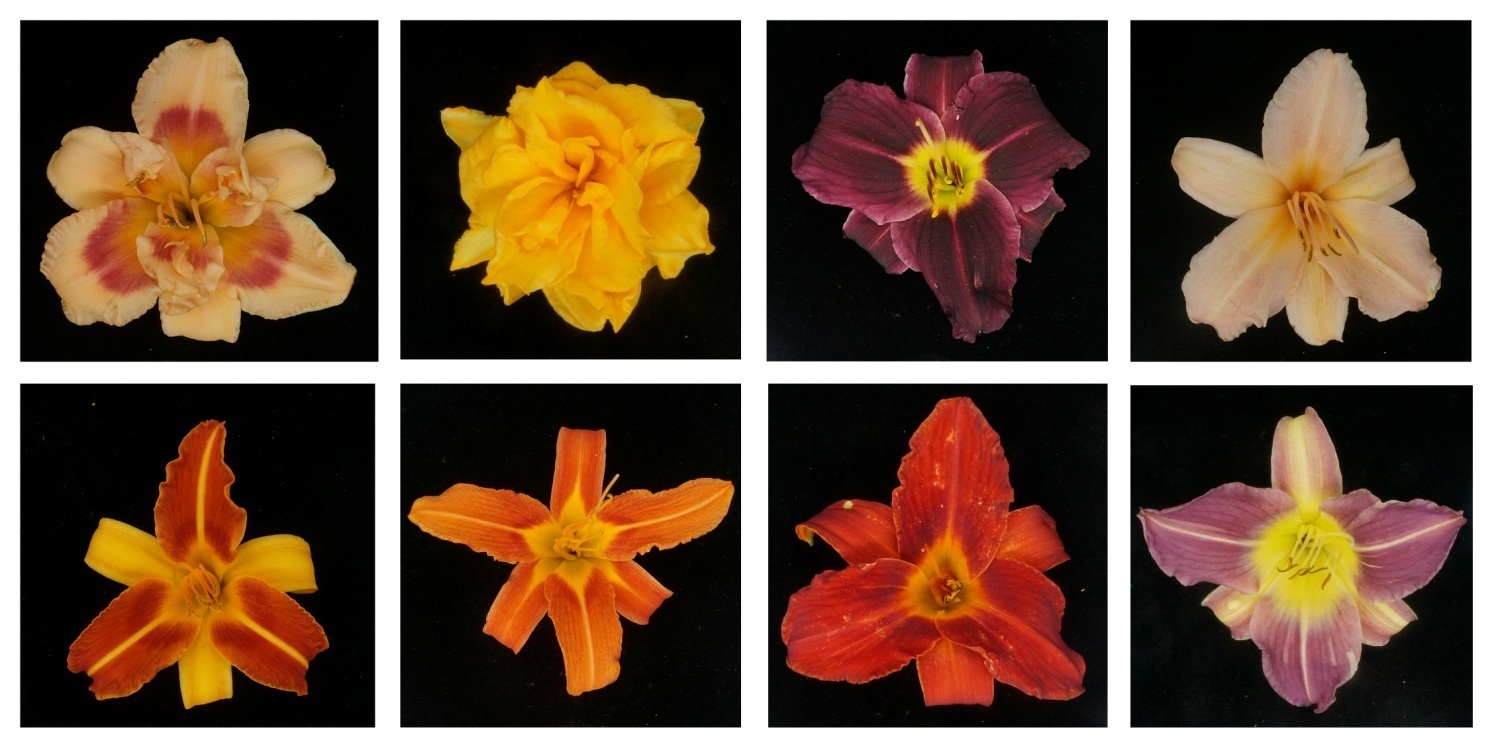


**Fig. S4** Diverse *Hemerocallis* varieties all sharing the same chloroplast genome (after removing gaps in the inter-gene region of *trnT-UGU* and *trnL-UAA*). Figures . (A) Forty Second Street (S207), (B) Betty Woods (S70), (C) Purple Waters (S87), (D) Childrens Festival (S76), (E) Frans Hals (S140), (F) Blazing Sun (S60), (G) Bourbon Kings (S81), (H) Blue Sheen (S62). All of the photos were taken in the experimental orchard located at Shanxi Agriculture University.

# Supplementary Tables

**Table S1** Information on 74 *Hemerocallis* samples.

| **Accession** | **NCBI** | **Taxonomy** | **Wild/Land race/Cultivar** | | **Lines name** | **Sample Source** | **Blooming Time** |
| --- | --- | --- | --- | --- | --- | --- | --- |
| S2 | OR260179 | *H. citrina* | | Landrace | Chazihua | Shaoyang, Hunan, China | Nocturnal |
| S3 | OR260106 | *H. citrina* | | Landrace | Qiezi 1 | Suzhou, Jiangsu, China | Nocturnal |
| S4 | OR260107 | *H. sp.* | | Cultivar | Panlonghua | Jinyun, Zhejiang, China | Nocturnal |
| S5 | OR260108 | *H. citrina* | | Landrace | Liuyuehua | Shaoyang, Hunan, China | Nocturnal |
| S6 | NC_064967.1 | *H. citrina* | | Landrace | Datonghuanghua | Datong, Shanxi, China | Nocturnal |
| S7 | OR260109 | *H. minor* | | Wild | *H. minor* | Qingyang, Gansu, China | Nocturnal |
| S8 | OR260110 | *H. lilioasphodelus* | | Wild | *H. lilioasphodelus* | Qingyang, Gansu, China | Nocturnal |
| S11 | OR260111 | *H. sp.* | | Cultivar | Guanglinghuanghua | Guangling, Shanxi, China | Nocturnal |
| S18 | OR260112 | *H. citrina* | | Landrace | Chonglihua 1 | Shaoyang, Hunan, China | Nocturnal |
| S20 | OR260113 | *H. citrina* | | Landrace | Dalihuanghua | Dali, Shanxi, China | Nocturnal |
| S21 | OR260114 | *H. citrina* | | Landrace | Baihua | Shaoyang, Hunan, China | Nocturnal |
| S22 | OR260115 | *H. sp.* | | Cultivar | Suqian 2 | Suqian, Jiangsu, China | Diurnal |
| S26 | OR260116 | *H. fulva* | | Wild | Lishan | Qinshui, Shanxi, China | Diurnal |
| S27 | OR260117 | *H. citrina* | | Landrace | Xianhuanghua 1 | Qingyang, Gansu, China | Nocturnal |
| S29 | OR260118 | *H. citrina* | | Landrace | Malinhuanghua | Qingyang, Gansu, China | Nocturnal |
| S30 | OR260119 | *H. citrina* | | Landrace | Huohuanghua | Qingyang, Gansu, China | Nocturnal |
| S31 | OR260120 | *H. citrina* | | Landrace | Gaotinghuanghua | Qingyang, Gansu, China | Nocturnal |
| S38 | OR260121 | *H. sp.* | | Cultivar | Taiguxuancao | Taigu, Shanxi, China | Diurnal |
| S39 | OR260122 | *H. citrina* | | Landrace | Dongzhuanghuanghua | Taigu, Shanxi, China | Nocturnal |
| S40 | OR260123 | *H.fulva* var. *aurantiaca* | | Wild | *H.fulva* var. *aurantiaca* | Haikou, Hainan, China | Diurnal |
| S41 | OR260124 | *H. middendorfii* | | Wild | *H. middendorfii* | Dailing, Heilongjiang, China | Diurnal |
| S45 | OR260125 | *H. multiflora* | | Wild | *H. multiflora* | Xinyang, Henan, China | Diurnal |
| S46 | OR260126 | *H. citrina* var. *altissima* | | Wild | *H. citrina* var. *altissima* | United States | Nocturnal |
| S47 | OR260127 | *H. citrina* | | Wild | Ankanghuanghuacai | Ankang, Shanxi, China | Nocturnal |
| S54 | OR260128 | *H. sp.* | | Cultivar | Autumn Red | United States | Diurnal |
| S56 | OR260129 | *H. sp.* | | Cultivar | Rocket City | Belgium | Diurnal |
| S58 | OR260130 | *H. sp.* | | Cultivar | Pink Damask | Belgium | Diurnal |
| S60 | OR260131 | *H. sp.* | | Cultivar | Blazing Sun | United States | Diurnal |
| S62 | OR260132 | *H. sp.* | | Cultivar | Blue Sheen 1 | Belgium | Diurnal |
| S63 | OR260133 | *H. sp.* | | Cultivar | Xiao Hong 74 | Baoding, Hebei, China | Diurnal |
| S70 | OR260134 | *H. sp.* | | Cultivar | Betty Woods | United States | Diurnal |
| S72 | OR260135 | *H. sp.* | | Cultivar | H400 | Baoding, Hebei, China | Diurnal |
| S74 | OR260136 | *H. sp.* | | Cultivar | Z-300 | Baoding, Hebei, China | Diurnal |
| S75 | OR260137 | *H. sp.* | | Cultivar | Little Wine Cup | Belgium | Diurnal |
| S76 | OR260138 | *H. sp.* | | Cultivar | Childrens Festival | Belgium | Diurnal |
| S80 | OR260139 | *H. sp.* | | Cultivar | Ruby | United States | Diurnal |
| S81 | OR260140 | *H. sp.* | | Cultivar | Bourbon King | Belgium | Diurnal |
| S83 | OR260141 | *H. sp.* | | Cultivar | Dong Fang Bu Bai | Baoding, Hebei, China | Diurnal |
| S84 | OR260142 | *H. sp.* | | Cultivar | Hong Bao | Baoding, Hebei, China | Diurnal |
| S85 | OR260143 | *H. sp.* | | Cultivar | XiaRi Jiu Hong | Baoding, Hebei, China | Diurnal |
| S87 | OR260144 | *H. sp.* | | Cultivar | Purple Waters | Belgium | Diurnal |
| S89 | OR260145 | *H. sp.* | | Cultivar | Orange | The Netherlands | Diurnal |
| S92 | OR260146 | *H. sp.* | | Cultivar | Xue Qiu Hong | Baoding, Hebei, China | Diurnal |
| S96 | OR260147 | *H. sp.* | | Cultivar | Beijing 1 | Beijing, China | Diurnal |
| S106 | OR260148 | *H. citrina* | | Landrace | Changzuizihua 2 | Qidong, Hunan, China | Diurnal |
| S107 | OR260149 | *H. citrina* | | Landrace | Xianjuhuanghua | Xianju, Zhejiang, China | Nocturnal |
| S109 | OR260150 | *H. citrina* | | Landrace | Gulaowuminghua | Qidong, Hunan, China | Nocturnal |
| S112 | OR260151 | *H. citrina* | | Landrace | Heizuizihua | Qidong, Hunan, China | Nocturnal |
| S113 | OR260152 | *H. citrina* | | Landrace | Zaohuanghua | Qidong, Hunan, China | Nocturnal |
| S115 | OR260153 | *H. citrina* | | Landrace | Chashantiaozhua | Qidong, Hunan, China | Nocturnal |
| S116 | OR260154 | *H. citrina* | | Landrace | Bayuehua | Qidong, Hunan, China | Nocturnal |
| S117 | OR260155 | *H. citrina* | | Landrace | Tianguanghua | Qidong, Hunan, China | Nocturnal |
| S118 | OR260156 | *H. sp.* | | Cultivar | Longyouhonghua | Longyou, Zhejiang, China | Diurnal |
| S121 | OR260157 | *H. citrina* | | Landrace | Dingzhuangdacai | Siyang, Jiangsu, China | Nocturnal |
| S122 | OR260158 | *H. citrina* | | Landrace | Siyuebai | Qidong, Hunan, China | Nocturnal |
| S123 | OR260159 | *H. citrina* | | Landrace | Quxianhuanghua | Quxian, Sichuan ,China | Nocturnal |
| S126 | OR260160 | *H. citrina* | | Landrace | Shayuanjinzhen | Dali, Shanxi, China | Nocturnal |
| S127 | OR260161 | *H. citrina* | | Landrace | Siyuehonghua | Qidong, Hunan, China | Nocturnal |
| S128 | OR260162 | *H. citrina* | | Landrace | Mengzihua | Qidong, Hunan, China | Nocturnal |
| S129 | OR260163 | *H. citrina* | | Landrace | Wupingzao | Sichuan, China | Nocturnal |
| S130 | OR260164 | *H. citrina* | | Landrace | Jingzhouhua | Qidong, Hunan, China | Nocturnal |
| S134 | OR260165 | *H. citrina* | | Landrace | Daojianhua | Qidong, Hunan, China | Nocturnal |
| S136 | OR260166 | *H. citrina* | | Landrace | Dawuzui | Jiangsu, China | Nocturnal |
| S137 | OR260167 | *H. citrina* | | Landrace | Xiyezihua | Qidong, Hunan, China | Nocturnal |
| S140 | OR260168 | *H. sp.* | | Cultivar | Frans Hals | The Netherlands | Diurnal |
| S141 | OR260169 | *H. sp.* | | Cultivar | Little Bumble Bee | Dongyang, Shanxi, China | Diurnal |
| S144 | OR260170 | *H. sp.* | | Cultivar | X-91 | Baoding, Hebei, China | Diurnal |
| S151 | OR260171 | *H. citrina* | | Landrace | Qingzaohua | Hunan, China | Nocturnal |
| S154 | OR260172 | *H. citrina* | | Landrace | Gaoganzhongqihua | Hunan, China | Nocturnal |
| S179 | OR260173 | *H. citrina* | | Landrace | Yanchihuanghua | Yanchi, Gansu, China | Nocturnal |
| S182 | OR260174 | *H. sp.* | | Landrace | Wennan 1 | Wennan, Henan, China | Nocturnal |
| S198 | OR260175 | *H. sp.* | | Cultivar | NA | Beijing, China | Diurnal |
| S199 | OR260176 | *H. sp.* | | Cultivar | NA | Beijing, China | Diurnal |
| S207 | OR260177 | *H. sp.* | | Cultivar | Forty Second Street | United States | Diurnal |

**Table S2** Annotated genes grouped by function and abbreviated accordingly.

| **Function** | **Gene Names** |
| --- | --- |
| Photosystem I | *psaB psaA psaI psaJ psaC* |
| Photosystem II | *psbA psbK psbI psbM psbD psbC psbZ psbJ psbL psbF psbE psbB psbT psbN psbH* |
| Cytochrome b/f complex | *petN petA petL petG petB petD* |
| ATP synthase | *atpA atpF atpH atpI atpE atpB* |
| NADH dehydrogenase | *ndhJ ndhK ndhC ndhB*2 ndhH ndhA ndhI ndhG ndhE ndhD ndhF* |
| RubisCO large subunit | *rbcL* |
| RNA polymerase | *rpoC2 rpoC1 rpoB rpoA* |
| Ribosomal proteins (SSU) | *rps12*2 rps16 rps2 rps14 rps4 rps18 rps11 rps8 rps3 rps19*2 rps7*2 rps15* |
| Ribosomal proteins (LSU) | *rpl33 rpl20 rpl36 rpl14 rpl16 rpl22 rpl2*2 rpl23*2 rpl32* |
| Hypothetical chloroplast reading frames | *ycf3 ycf4 ycf2*2 ycf1* |
| Translation initiation factor IF-1 | *infA* |
| Acetyl-CoA carboxylase | *accD* |
| Cytochrome c biogenesis Maturase | *matK* |
| ATP-dependent protease | *clpP* |
| Inner membrane protein | *cemA* |
| Ribosomal RNAs | *rrn16*2 rrn23*2 rrn4.5*2 rrn5*2* |
| Transfer RNAs | *trnA-UGC*2 trnC-GCA trnD-GUC trnE-UUC trnF-GAA trnG-GCC trnG-UCC trnH-GUG*2 trnI-GAU*2 trnK-UUU trnL-CAA*2 trnL-UAA trnL-UAG trnM-CAU*4 trnN-GUU*2 trnP-UGG trnQ-UUG trnR-ACG*2 trnR-UCU trnS-GCU trnS-GGA trnS-UGA trnT-GGU trnT-UGU trnV-GAC*2 trnV-UAC trnW-CCA trnY-GUA* |
| *2 : Two copies |  |

**Table S3** Characteristics of circularized chloroplast genomes.

| Accession | Length of genome (bp) | Length of SSC (bp) | Length of IR (bp) | Length of LSC (bp) | Length of Intergenetic region between *trnT-UGU* and *trnL-UAA* | GC content of whole genomes | GC content of Intergenetic region between *trnT-UGU* and *trnL-UAA* |
| --- | --- | --- | --- | --- | --- | --- | --- |
| S6 | 156083 | 84838 | 26369 | 18507 | 1209bp | 37.50% | 17.37% |
| S118 | 156076 | 84831 | 26369 | 18507 | 1207bp | 37.50% | 17.40% |
| S11 | 156075 | 84830 | 26369 | 18507 | 1210bp | 37.50% | 17.27% |
| S121 | 156110 | 84841 | 26369 | 18531 | 1200bp | 37.50% | 17.33% |
| S18 | 156110 | 84841 | 26369 | 18531 | 1200bp | 37.50% | 17.33% |
| S127 | 156052 | 84804 | 26369 | 18510 | 1204bp | 37.50% | 17.44% |
| S129 | 156068 | 84829 | 26369 | 18501 | 1204bp | 37.50% | 17.44% |
| S179 | 156088 | 84843 | 26369 | 18507 | 1210bp | 37.50% | 17.36% |
| S29 | 156088 | 84843 | 26369 | 18507 | 1210bp | 37.50% | 17.36% |
| S39 | 156086 | 84841 | 26369 | 18507 | 1210bp | 37.50% | 17.36% |

**Table S4** Accessions of duplicates sharing the same genome sequence (after removing gaps in the inter-gene region of *trnT-UGU* and *trnL-UAA*)

| Number of duplicates | Accessions with same genome | | | | | | | | | | | | | | | | | | |
| --- | --- | --- | --- | --- | --- | --- | --- | --- | --- | --- | --- | --- | --- | --- | --- | --- | --- | --- | --- |
| 19 | S107 | S115 | S117 | S123 | S126 | S130 | S134 | S137 | S179 | S199 | S20 | S27 | S29 | S2 | S39 | S3 | S4 | S5 | S7 |
| 15 | S140 | S141 | S144 | S207 | S47 | S60 | S62 | S63 | S70 | S72 | S74 | S76 | S81 | S84 | S87 |  |  |  |  |
| 4 | S40 | S56 | S85 | S89 |  |  |  |  |  |  |  |  |  |  |  |  |  |  |  |
| 3 | S21 | S30 | S31 |  |  |  |  |  |  |  |  |  |  |  |  |  |  |  |  |
| 3 | S113 | S122 | S151 |  |  |  |  |  |  |  |  |  |  |  |  |  |  |  |  |
| 3 | S121 | S154 | S18 |  |  |  |  |  |  |  |  |  |  |  |  |  |  |  |  |
| 2 | S116 | S128 |  |  |  |  |  |  |  |  |  |  |  |  |  |  |  |  |  |
| 2 | S54 | S83 |  |  |  |  |  |  |  |  |  |  |  |  |  |  |  |  |  |
| 2 | S106 | S8 |  |  |  |  |  |  |  |  |  |  |  |  |  |  |  |  |  |
|  | | | | | | | | | | | | | | | | | | | |

**Table S5** The top ten sites with the highest Pi values on the chloroplast genome and their corresponding Pi values. The underscore indicates the intergenic region.

| **Location of peak** | **Pi value** |
| --- | --- |
| *rpl32_trnL-UAG* | 0.0176 |
| *rpl32* | 0.00962 |
| *psaA_ycf3* | 0.00954 |
| *rps15* | 0.00911 |
| *ycf3* | 0.00907 |
| *rps15_ycf1* | 0.00827 |
| *ndhF_rpl32* | 0.00777 |
| *psbE_petL* | 0.00765 |
| *psaC_ndhE* | 0.00722 |
| *trnS-GGA_trnG-UCC* | 0.00663 |

**Table S6** Information on the variant sites that appear only in the daylily group, using S129 (nightlily, nocturnal) as the reference sequence.

| **Location on the reference (bp)** | **Variants Sequence** | **Gene** | **Polymorphism Type** | **Variant Sequences** |
| --- | --- | --- | --- | --- |
| 45419 | G -> T | - | SNP (transversion) | OR260114, OR260115, OR260116, OR260121, OR260123, OR260125, OR260128, OR260130, OR260137, OR260146, OR260147, OR260156, OR260174 |
| 47769 | (A)10 -> (A)11 | - | Insertion (tandem repeat) | OR260114, OR260115, OR260116, OR260121, OR260123, OR260125, OR260128, OR260130, OR260137, OR260146, OR260147, OR260156, OR260174 |
| 56285 | T -> G | - | SNP (transversion) | OR260114, OR260115, OR260116, OR260121, OR260123, OR260125, OR260128, OR260130, OR260137, OR260146, OR260147, OR260156, OR260174 |
| 61027 | C -> T | *ycf4* | SNP (transition) | OR260114, OR260115, OR260116, OR260121, OR260123, OR260125, OR260128, OR260130, OR260137, OR260146, OR260147, OR260156, OR260174 |
| 64253 | -CCAAA | *-* | Deletion | OR260114, OR260115, OR260116, OR260121, OR260123, OR260125, OR260128, OR260130, OR260137, OR260146, OR260147, OR260156, OR260174 |
| 75759 | T -> G | *-* | SNP (transversion) | OR260114, OR260115, OR260116, OR260121, OR260123, OR260125, OR260128, OR260130, OR260137, OR260146, OR260147, OR260156, OR260174 |
| 76045 | G -> T | *-* | SNP (transversion) | OR260114, OR260115, OR260116, OR260121, OR260123, OR260125, OR260128, OR260130, OR260137, OR260146, OR260147, OR260156, OR260174 |
| 82134 | T -> G | *-* | SNP (transversion) | OR260114, OR260115, OR260116, OR260121, OR260123, OR260125, OR260128, OR260130, OR260137, OR260146, OR260147, OR260156, OR260174 |
| 92447 | A -> C | *ycf2* | SNP (transversion) | OR260114, OR260115, OR260116, OR260121, OR260123, OR260125, OR260128, OR260130, OR260137, OR260146, OR260147, OR260156, OR260174 |
| 118763 | G -> A | *-* | SNP (transition) | OR260114, OR260115, OR260116, OR260121, OR260123, OR260125, OR260128, OR260130, OR260137, OR260146, OR260147, OR260156, OR260174 |
| 120235 | G -> T | *-* | SNP (transversion) | OR260114, OR260115, OR260116, OR260121, OR260123, OR260125, OR260128, OR260130, OR260137, OR260146, OR260147, OR260156, OR260174 |
| 125019 | G -> T | *-* | SNP (transversion) | OR260114, OR260115, OR260116, OR260121, OR260123, OR260125, OR260128, OR260130, OR260137, OR260146, OR260147, OR260156, OR260174 |
| 127133 | T -> G | *ycf1* | SNP (transversion) | OR260114, OR260115, OR260116, OR260121, OR260123, OR260125, OR260128, OR260130, OR260137, OR260146, OR260147, OR260156, OR260174 |
| 148451 | T -> G | *ycf2* | SNP (transversion) | OR260114, OR260115, OR260116, OR260121, OR260123, OR260125, OR260128, OR260130, OR260137, OR260146, OR260147, OR260156, OR260174 |

**Table S7** Taxanomic information of outgroup species

| **Species** | **Genus** | **Family** | **Order** | **Genbank accession number** |
| --- | --- | --- | --- | --- |
| *Eremurus robustus* | *Eremurus* | Asphodelaceae | Asparagales | NC 046772 |
| *Xanthorrhoea preissii* | *Xanthorrhoea* | Asphodelaceae | Asparagales | NC 035996 |
| *Aloe vera* | *Aloe* | Asphodelaceae | Asparagales | NC_035506 |
| *Allium chinense* | *Allium* | Amaryllidaceae | Asparagales | NC 043922.1 |

**Table S8** Trait codes for ancestral state reconstruction

| **Accesion** | **Species** | **Flowering Time** | **Trait Code** | **Color of Petals** | **Trait Code** |
| --- | --- | --- | --- | --- | --- |
| S6 | *Hemerocallis citrina* cv. *‘datonghuanghua’* | Nocturnal | 1 | Yellow | 0 |
| S7 | *Hemerocallis minor* | Nocturnal | 1 | Yellow | 0 |
| S8 | *Hemerocallis lilioasphodelus* | Nocturnal | 1 | Yellow | 0 |
| S27 | *Hmerocallis fulva* | Diurnal | 0 | Reddish orange | 3 |
| S40 | *Hemerocallis fulva* var. *aurantiaca* | Diurnal | 0 | Orange yellow | 2 |
| S41 | *Hemerocallis middendorfii* | Diurnal | 0 | Orange yellow | 2 |
| S45 | *Hemerocallis multiflora* | Diurnal | 0 | Gold | 1 |
| S47 | *Hemerocallis citrina* var. *altissima* | Nocturnal | 1 | Yellow | 0 |
| S128 | *Hemerocallis citrina* cv. *‘mengzihua’* | Nocturnal | 1 | Yellow | 0 |
